# Effect of different form of upper limb muscles training on dyspnea in chronic obstructive pulmonary disease: A study protocol

**DOI:** 10.1097/MD.0000000000022131

**Published:** 2020-09-18

**Authors:** Marc Beaumont, Loic Péran, Anne Cécile Berriet, Catherine Le Ber, Patricia Le Mevel, Emmanuelle Courtois-Communier, Francis Couturaud

**Affiliations:** a Pulmonary Rehabilitation Unit, Morlaix Hospital Centre, Morlaix; b EA3878 (Groupe d’Etude de la Thrombose de Bretagne Occidentale); c INSERM Centre d’Investigation Clinique 1412, University Hospital of Brest; d Department of Internal Medicine and Chest Diseases, EA3878 (GETBO), CIC INSERM 1412, University Hospital of Brest, European University of Occidental Brittany, Brest, France.

**Keywords:** activities of daily living, chronic obstructive pulmonary disease, dyspnea, pulmonary rehabilitation, strengthening, upper limb

## Abstract

**Introduction:**

In activities involving upper limbs, patients with chronic obstructive pulmonary disease (COPD) report an increase in dyspnea. For this reason, the authors of the recommendations about pulmonary rehabilitation propose to perform upper limbs muscle strengthening in patients with COPD. However, the modalities of strengthening are not clearly established.

The aim of this study is to compare the effects of upper limbs endurance strengthening versus upper limbs force strengthening, in patients with COPD during a pulmonary rehabilitation program.

**Methods:**

This study is a randomized, open-label, bi-center controlled trial in parallel groups distributed in a ratio (1:1) comparing upper limbs force strengthening (group F) to the upper limbs endurance strengthening (group E) during a pulmonary rehabilitation program in patients with COPD stages 2 to 4 (A–D).

After randomization, patients will be allocated to follow:

A 4 weeks pulmonary rehabilitation program with upper limbs resistance strengthening (group F).

A 4 weeks pulmonary rehabilitation program with upper limbs endurance strengthening (group E).

The primary outcome is dyspnea measured with the London Chest Activity of daily Living questionnaire. The secondary outcomes are dyspnea (using Modified Medical Research Council dyspnea Scale, dyspnea-12 questionnaire, multidimensional dyspnea profile questionnaire), upper limb exercise capacity (using the 6-minute Peg Board and Ring Test), Maximal voluntary strength of deltoid, biceps, and brachial triceps.

The sample size calculated is 140 patients per group, or 280 in total.

**Discussion:**

The modalities of upper limb strengthening are not very well known, and evidence based is lacking to recommend endurance or resistance upper limb strengthening.

We anticipate that the results of this study will be of relevance to clinical practice. They will bring information about the best modality of upper limb strengthening to use during a pulmonary rehabilitation program.

**Trial registration:**

IdRCB n°2018-A00955-50; V1.1 du 11/07/2018; REHABSUP, clinical trial.gov (NCT03611036), registered August 02, 2018, https://clinicaltrials.gov/ct2/show/NCT03611036.

## Abstract 2

Supplemental Digital Content is available in the text

## 1 Introduction

Pulmonary rehabilitation is a cardinal component of chronic obstructive pulmonary disease (COPD) management.^[1,2]^ During the past decade, several prospective trials have shown that pulmonary rehabilitation in COPD patients was associated with a decrease in COPD-related handicap and an improvement of quality of life. In addition, a reduction of mortality was suggested in one of these studies when pulmonary rehabilitation was performed early after an acute COPD exacerbation.^[3,4]^ The pulmonary rehabilitation program includes individualized exercise training, therapeutic education, respiratory physiotherapy, help with smoking cessation, and nutritional and psychosocial coverage. Exercise training must include lower-limb training associated with upper-limb training. Indeed, many patients with COPD report difficulties in performing activities using upper limbs due to the dyspnea generated by these movements and upper limb fatigue.^[5–7]^ In addition, activities using upper limbs are performed at a lower intensity compared with healthy subjects.^[8]^ In COPD patients, the difficulties encountered in performing upper limb activities are partly related to respiratory disease, and in particular to the fact that the muscles required to mobilize the upper limbs are also required for ventilatory mechanics.^[6]^ Tangri and Woolf^[9]^ have shown that COPD patients have difficulties in activities of daily living (ADL) using upper limbs, especially for combing their hair, brush their teeth. They observed a modification of the respiratory pattern, breathing becomes rapid, shallow, and irregular.

Therefore, in activities involving upper limbs, patients with COPD report an increase in dyspnea. Upper limbs are used for 80% of daily life activities, upper limbs strengthening seems important to decrease dyspnea in patients with COPD.^[10]^

So, for these reasons, the authors of the recommendations about pulmonary rehabilitation propose to perform upper limbs muscle strengthening in patients with COPD.^[1]^ The objectives of this strengthening are to reduce dyspnea, improve quality of life, and improve strength and endurance of the upper limbs. However, the methods of muscle strengthening are not specified. The authors of a recent systematic review with meta-analysis^[11]^ report that during a pulmonary rehabilitation program, upper limbs exercise training decreases dyspnea, in comparison with no upper limbs exercise training. They mention that the modalities of strengthening are not clearly established, and they conclude that studies are necessary for clarifying modalities of strengthening in comparing upper limbs endurance strengthening versus upper limbs force strengthening.

The aim of this study is to compare the effects of upper limbs endurance strengthening versus upper limbs force strengthening, in patients with COPD during a pulmonary rehabilitation program.

## 2 Methods/Design

### 2.1 Setting

This prospective bi-center, open label randomized trial will be conducted in 2 pulmonary rehabilitation units.

### 2.2 Population

Patients routinely admitted for a pulmonary rehabilitation program (4 weeks) in Centre Hospitalier des Pays de Morlaix (Morlaix, France) or in Centre Hospitalier Universitaire de Brest (Brest, France) will be included for the study if they had COPD stages from 2 to 4 (A–D) diagnosed according to GOLD criteria^[12]^ at admission (Forced Expiratory Volume in 1 second/Forced Vital Capacity < 0.7, Forced Expiratory Volume in 1 second < 80% of predicted value), if they are at least 18 years old, and if they sign consent form after receiving written information.

Non-inclusion criteria will be:

- Patient with shoulder pain, shoulder osteoarthritis, or shoulder surgery.
- Previous pneumonectomy or lobectomy in the past 6 months.
- Refusal of participation.
- Patient with incapacity to follow a standard rehabilitation program.
- Pregnant or breast-feeding women.
- Patient under tutorship or curatorship.

### 2.3 Study design

Participation in the study will be considered for all COPD patients admitted to the pulmonary rehabilitation department of the Centre Hospitalier des Pays de Morlaix or Centre Hospitalier Universitaire de Brest.

Eligible patients will be included after the investigator has provided complete oral and written information to the patients, completed the initial assessments for the pulmonary rehabilitation program, verified the inclusion and non-inclusion criteria, and obtained written consent form from patients.

The initial assessments for the pulmonary rehabilitation program include the following tests:

- Spirometry with Forced Expiratory Volume in 1 second and Forced Vital Capacity maneuvers after bronchodilators (spirometry with plethysmography will be performed during the pulmonary rehabilitation program).
- Arterial puncture is performed for arterial blood gases analysis.
- Inspiratory muscle strength (PImax) is measured using a Micro RPM (Micro Medical, Rochester, UK).
- Exercise capacity is measured using 6-minute walk test (6MWT), 1-minute Sit to Stand test (1STST), and maximal cardiopulmonary exercise test on cycloergometer.
- Maximal voluntary quadriceps strength and quadriceps endurance are measured using a hand held dynamometer.^[13,14]^
- Dyspnea is evaluated according the recommendations^[15]^ with Dyspnea-12 questionnaire,^[16]^ Modified Medical Research Council dyspnea Scale,^[17]^ London Chest Activity of daily Living (LCADL),^[10]^ multidimensional dyspnea profile (MDP)^[18]^ at the end of the 6MWT.
- Quality of life is measured with Saint George Respiratory questionnaire (SGRQ)^[19]^ and COPD Assessment Test (CAT).^[20]^
- Anxiety and depression are evaluated using Hospital Anxiety and Depression Scale (HAD-A and HAD-D).^[21]^
- Fear of falling is evaluated using Fall efficacy Scale-International (FES-I)^[22]^
- Educational interview for therapeutic education program is performed
- Consultation for smoking cessation and/or consultation with a nutritionist is realized if necessary.

Others tests will be performed for this study:

- The 6-minute Peg Board and Ring Test (6PBRT):
- Maximal voluntary strength of deltoid, biceps, and brachial triceps are measured using a hand held dynamometer.

### 2.4 Randomization and allocation procedure

This study is a Randomized, open-label, bi-centre controlled trial in parallel groups distributed in a ratio (1:1) comparing upper limbs force strengthening (group F) to the upper limbs endurance strengthening (group E) during a pulmonary rehabilitation program in patients with COPD stages 2 to 4 (A–D).

Block randomization are performed with variable block sizes. Block sizes are randomly established by the statistician. Investigators will be kept blinded to each block size, to ensure that they could not become aware of patients’ allocation in advance.

After randomization, patients will be allocated to follow:

- A 4 weeks pulmonary rehabilitation program with upper limbs force strengthening (group F).
- A 4 weeks pulmonary rehabilitation program with upper limbs endurance strengthening (group E).

Primary endpoint is open-label because it is an auto-questionnaire (LCADL). The other endpoints will be blinded evaluated, with an assessor, which will not participate to inclusion, randomization, nor pulmonary rehabilitation program.

### 2.5 Intervention

The standardized pulmonary rehabilitation program (both groups) will be conducted over 4 weeks, 5 days per week and will include aerobic exercise on a cycle ergometer and a treadmill (each for 30 minutes per day),^[9,10]^ strengthening of lower limb muscle groups, a therapeutic educational program, aerobic gymnastics in groups, a smoking cessation program, and sociopsychological and dietary advice.

Randomized intervention for group E:

- In group E, patients will perform in addition to the classic pulmonary rehabilitation program, upper limbs endurance strengthening.

Details of upper limbs endurance strengthening:

- It will be performed 3 times a week for 4 weeks, supervised by a physiotherapist. This strengthening is performed with dumbbells whose weight is determined according to the strength of the muscles of the upper limbs evaluated at the beginning of the program (at 30% of the maximum voluntary force).

Description of the exercises:

- The patients have to realize 4 exercises. Patients are sitting on a stool.
- Exercise 1: upper limbs along the body => bending of the elbows then extending the elbows along the body.
- Exercise 2: hands to shoulders => extension of the elbows to the front then back of the hands to the shoulders.
- Exercise 3: hands to shoulders => elbow extension up, then hand back to shoulders.
- Exercise 4: upper limbs along the body => abduction of the upper limbs to the horizontal (elbows stretched), then back of the upper limbs along the body.
- Maximum 1-minute rest between each exercise.

Description of the sessions during the 4 weeks

Week 1:

- Weight of the dumbbells at 30% of the maximum voluntary force.
- Perform 3 series of 10 movements for each exercise (exercise 1 to exercise 4).

Week 2:

- Weight of the dumbbells at 30% of the maximum voluntary force.
- Perform 3 series of 15 movements for each exercise (exercise 1 to exercise 4).

Week 3:

- Weight of the dumbbells at 30% of the maximum voluntary force.
- Perform 3 series of 20 movements for each exercise (exercise 1 to exercise 4).

Week 4:

- Weight of the dumbbells at 30% of the maximum voluntary force.
- Perform 3 series of 25 movements for each exercise (exercise 1 to exercise 4).

Randomized intervention for group F:

- In group F, patients will perform in addition to the classic pulmonary rehabilitation program, upper limbs force strengthening.

Details of upper limbs force strengthening:

- It will be performed 3 times a week for 4 weeks, supervised by a physiotherapist. This strengthening is performed with dumbbells whose weight is determined according to the strength of the muscles of the upper limbs evaluated at the beginning of the program (at 60% to 80% of the maximum voluntary force).

Description of the exercises:

- The patients have to realize 4 exercises. Patients are sitting on a stool.
- Exercise 1: upper limbs along the body => bending of the elbows then extending the elbows along the body.
- Exercise 2: hands to shoulders => extension of the elbows to the front then back of the hands to the shoulders.
- Exercise 3: hands to shoulders => elbow extension up, then hand back to shoulders.
- Exercise 4: upper limbs along the body => abduction of the upper limbs to the horizontal (elbows stretched), then back of the upper limbs along the body.
- Maximum 1-minute rest between each exercise.

Description of the sessions during the 4 weeks

Week 1:

- Weight of the dumbbells at 60% of the maximum voluntary force.
- Perform 2 series of 10 movements for each exercise (exercise 1 to exercise 4).

Week 2:

- Weight of the dumbbells at 70% of the maximum voluntary force.
- Perform 2 series of 10 movements for each exercise (exercise 1 to exercise 4).

Week 3:

- Weight of the dumbbells at 80% of the maximum voluntary force.
- Perform 2 series of 10 movements for each exercise (exercise 1 to exercise 4).

Week 4:

- Weight of the dumbbells at 80% of the maximum voluntary force.
- Perform 3 series of 10 movements for each exercise (exercise 1 to exercise 4).

### 2.6 Outcomes

#### 2.6.1 Primary outcome

London Chest Activity of daily Living questionnaire (LCADL).^[10]^

This 15-item, self-administered questionnaire has been developed by Garrod et al.^[10,23]^ It allows an evaluation of dyspnea in patients with COPD during daily activities divided into 4 components: self-care, domestic, physical, and leisure. Patients could score from 0: “I would not do anyway” to 5: “I need someone else to do this.” LCADL score is calculated by aggregating the points assigned to each question (0–75), with a higher score representing maximal disability. MID has been determined for LCADL.^[24,25]^ A decrease of 4 points during a pulmonary rehabilitation program is clinically important for patients.

#### 2.6.2 Secondary outcomes

Dyspnea will be also assessed with other scales: Modified Medical Research Council dyspnea scale^[17]^ and Dyspnea-12 questionnaire which allows to assess sensory and emotional components of dyspnea.^[16,26]^

The 6-minute Peg Board and Ring Test (6PBRT):

- The 6PBRT is a good test to evaluate upper limbs exercise capacity in COPD patients, and Takeda et al^[27]^ showed a correlation between 6PBRT score and physical activity. In our study, we will use 6PBRT as a functional marker, in order to measure upper limbs endurance.
- The 6PBRT was performed according to the method of Zhan et al,^[28]^ with a slight modification.

Patients sit in a chair and a pegboard with multiple peg positions is placed in front of the subject at arm's length from the body. Two pegs are positioned at the shoulder level and 2 at 20 cm above the shoulder level, and 5 rings (Zhan used 10 rings) (15 g per ring) are put on each of the 2 lower pegs. Patients are instructed to use both hands simultaneously to move 1 ring from each of the lower pegs to the upper pegs: subjects need to use the left hand to move the ring on the left lower peg, and the right hand to move the ring on the right lower peg. After all 10 rings are moved from the lower pegs to the upper pegs, patients move again 1 ring from each of the upper pegs back to the lower pegs. Before the test, patients are allowed to do 1 cycle of moving up and down as a practice to familiarize themselves with the procedures. At the signal “go,” subjects need to move as many pegs as possible during 6-minute. The final score is the total number of rings moved during the 6-minute period. Subjects are permitted to stop and rest during the test if they feel severe dyspnea, fatigue, or other discomfort, and continue moving the pegs as soon as they can. The patient is encouraged every minute using the standard phrases used during the 6MWT.

Subjects were asked to score the perceived dyspnea and upper limb fatigue before and at the end of the test by using Borg scale. Each patient will perform the 6PBRT twice. A pulse oximeter will be used to monitor heart rate (HR) and pulse oxygen saturation (Spo2) at the beginning and at the end of the test.

To avoid the potential problem of fatigue, a 30-minute resting interval will be given between the 2 6PBRT. Heart rate, Spo2, dyspnea an upper limb fatigue measures will be used to determine if patient’ physiologic status return to the baseline level. If not, a longer resting interval will be given to the patient.

Maximal voluntary strength of deltoid, biceps, and brachial triceps:

- Maximal voluntary strength is measured using a handheld dynamometer, MicroFET 2 (Hoggan Health BIOMETRICS France – 40 A 42 - 40, Route de Chartres – 91940 Gometz-Le-Châtel).

Measure of Maximal voluntary strength of deltoid:

- Maximal voluntary strength of deltoid will be measured in semi-seated position: patient is in supine position with the trunk at 45° relative to the horizontal, shoulder at 90° abduction resting on the table, elbow in flexion. The microfet is placed above the elbow joint.
- The assessor asks the patient to spread the arm as strongly as possible.
- Both upper limbs are tested.
- The instructions given are: “The objective of this test is to measure the strength of the shoulder muscle. You will spread the arm as strongly as you can and maintain the contraction for 1 to 3 seconds. I will tell you when you need to push. I will encourage you. There will be 3 trials.”

Measure of Maximal voluntary strength of biceps:

- The patient is supine, trunk 45° from horizontal, arm resting on the table, and elbow bent 90°. The Microfet is placed on the front of the distal end of the forearm.
- The assessor asks the patient to bend the elbow as strongly as possible.
- Both upper limbs are tested.
- The instructions given are: “The objective of this test is to measure the strength of the biceps muscle. You will bend the elbow as strongly as you can and maintain the contraction for 1 to 3 seconds. I will tell you when you will have to bend. I will encourage you. There will be 3 trials.”

Measure of Maximal voluntary strength of brachial triceps:

- The patient is supine, trunk 45° from horizontal, arm resting on the table, and elbow bent 90°. The Microfet is positioned on the posterior side of the distal end of the forearm.
- Both upper limbs are tested.
- The instructions given are: “The objective of this test is to measure the strength of the arm muscle. You will extend your elbow as strongly as you can and maintain the contraction for 1 to 3 seconds. I will tell you when you will have to extend. I will encourage you. There will be 3 trials.”
- For each muscle, 3 tests are performed on each upper limb, with a 1-minute rest period between each contraction.
- The variation between the values obtained must not exceed 10%.
- The best value is kept as the maximum voluntary strength value.

### 2.7 Sample size calculation

A previous study^[29]^ (EMI2) reported a standard deviation of 10 points for the LCADL score. A 4-point improvement for LCADL during a pulmonary rehabilitation program would be clinically significant.^[24,25]^

With these assumptions, for an α-error of 5% and β-error of 10%, the expected sample size would therefore be 133 patients per group. In order to anticipate loss to follow-up or withdrawals of consent, 140 patients per group will be included, or 280 in total.

### 2.8 Statistical analysis

All the data will be collected by a research nurse blinded to treatment allocation. Continuous variables will be expressed as mean ± SD, as median (interquartile range) or frequency.

The mean's evolution for the endpoints obtained in each group will be analyzed using a Student *t* test or Wilcoxon test (in case of non-normal distribution) for within-group and between-group comparisons, respectively.

All data will be analyzed in an intention-to-treat analysis.

A *P*-value of <.05 will be considered statistically significant.

### 2.9 Ethics and dissemination

The study was approved by the ethics board (CPP Ouest 4, n° 2018-A00955-50) and registered on clinical trial.gov (NCT03611036). Written informed consent will be obtained from all patients. The results arising from this randomized trial will be presented at scientific meetings as abstracts for poster or oral presentations and published in peer-reviewed journals. There is no intention of using a professional writer, and authorship will be based on the collaboration of each member of the research group.

### 2.10 Data monitoring

A Clinical Research Associate (ARC) appointed by the promotor (CHRU Brest) will ensure the successful completion of the study, the collection of the data generated in writing, their documentation, registration and report, in accordance with the Standard Operating Procedures implemented within the CHRU de Brest and in accordance with the Good Clinical Practices as well as the legal and regulatory provisions in force (Supplemental Digital Content, "dgos_resultats_phrip_2017_280618.xlsx": http://links.lww.com/MD/E846).

In the event of an adverse event, serious or not, follow-up will be considered in accordance with the usual management of patients of the unit.

## 3 Discussion

This randomized trial will compare 2 modalities of upper limbs strengthen during a pulmonary rehabilitation program. Outcomes will be dyspnea (primary outcome), strength, and endurance of upper limb muscles. Upper limb strengthening will be performed with dumbbells after an evaluation using a handheld dynamometer, which can objective the weight of the dumbbells for each patient according the recommendations.^[30]^

The modalities of upper limb strengthening are not very well known, and evidence based is lacking to recommend endurance or resistance upper limb strengthening.^[11]^

We anticipate that the results of this study will be of relevance to clinical practice. They will bring information about the best modality of upper limb strengthening to use during a pulmonary rehabilitation program.

## Author contributions

**Anne Cécile Berriet:** conception, design of the work, read and approved the submitted version.

**Catherine Le Ber:** conception, design of the work, read and approved the submitted version.

**Emmanuelle Courtois-Communier:** conception, design of the work, read and approved the submitted version.

**Francis Couturaud:** conception, design of the work, read and approved the submitted version.

**Loic Péran:** conception, design of the work, read and approved the submitted version.

**Marc Beaumont:** conception, design of the work, drafted the work, read and approved the submitted version.

**Patricia Le Mevel:** conception, design of the work, read and approved the submitted version.

## Abbreviations

Abbreviations: 1STST = 1-minute Sit to Stand test, 6MWT = 6-minute walk test, 6PBRT = 6-minute Peg Board and Ring Test, ADL = activities of daily living, CAT = COPD Assessment Test, COPD = chronic obstructive pulmonary disease, FES-I = Fall efficacy Scale- International, HAD-A and HAD-D = Hospital Anxiety and Depression Scale, LCADL = London Chest Activity of daily Living, MDP = multidimensional dyspnea profile, SGRQ = Saint George respiratory questionnaire.
